# Evaluation of prothrombotic risk of two *PROC* hotspot mutations (Arg189Trp and Lys193del) in Chinese population: a retrospective study

**DOI:** 10.1186/s12959-023-00548-6

**Published:** 2023-10-03

**Authors:** Lei Li, Jian Li, Xi Wu, Wenman Wu, Qiulan Ding, Baohua Qian, Xuefeng Wang

**Affiliations:** 1 Department of Laboratory Medicine, Ruijin Hospital, Shanghai Jiao Tong University School of Medicine, Shanghai, China; 2 State Key Laboratory of Medical Genomics, Shanghai Institute of Hematology, Ruijin Hospital, Shanghai Jiao Tong University School of Medicine, Shanghai, China; 3 Clinical Research Center, Ruijin Hospital, Shanghai Jiao Tong University School of Medicine, Shanghai, China; 4 Collaborative Innovation Center of Hematology, Shanghai Jiao Tong University School of Medicine, Shanghai, China; 5 Department of Transfusion Medicine, Changhai Hospital, Second Military Medical University, Shanghai, China

**Keywords:** Protein C deficiency, Hotspot mutation, Venous thromboembolism, Risk factors

## Abstract

**Background:**

R189W and K193del of protein C (PC) were hotspot mutations in Chinese population with venous thromboembolism (VTE), but almost two-thirds of patients with above mutations coexisting with other genetically or aquiredly prothrombotic risk factors. The aim of this study is to clarify the independent contributions of R189W or K193del to VTE risk.

**Methods:**

490 unrelated patients with a personal history of VTE and 410 healthy participants were enrolled in this study. Data of their demographics, family history, genetic and acquired thrombosis risk factors were collected and statistically analyzed.

**Results:**

PC R189W and K193del were identified in 3/410 (0.7%) and 7/410 (1.7%) healthy controls, and in 27/490 (5.5%) and 43/490 (8.8%) patients with VTE, respectively. Notably, about 70% of these mutant carriers combined with other genetic or acquired thrombophilic factors. After adjustment for age, gender, other inherited and acquired risk factors, we demonstrated that R189W and K193del were associated with 5.781-fold and 4.365-fold increased risk of VTE, respectively, which were significantly lower than the prothrombotic risk of anticoagulant deficiencies induced from rare mutations. Independent R189W or K193del mutation was not associated with earlier first-onset age as well as higher recurrent rate of VTE. However, combination of other genetic or acquired thrombophilic factors had supra-additive effects on those consequences. The more additional risk factors the patients had, the younger first-onset ages and higher risk of recurrence would be.

**Conclusions:**

As the most frequent mutations for PC deficiency in Chinese population, both R189W and K193del mutations had limited independent contributions to VTE development compared with other rare mutations in *PROC* gene, but may act in concert with other genetic defects or acquired thrombotic risk factors to produce the final severe phenotype.

**Supplementary Information:**

The online version contains supplementary material available at 10.1186/s12959-023-00548-6.

## Introduction

Venous thromboembolism (VTE) is a multifactorial disease caused by both acquired and genetic risk factors [1]. The main acquired disorders associated to VTE are antiphospholipid antibody syndrome (APS), hyperhomocysteinemia, transiently/permanently increased levels of coagulation factors or anticoagulants deficiency (surgery, trauma, pregnancy and hormonal therapy et al.) [2]. Genetic risk factors mainly include loss of function in anticoagulant factors (antithrombin (AT), protein C (PC) and protein S (PS)), or gain of function in procoagulants such as factor V Leiden and prothrombin G20210A [3–6]. However, the prothrombotic risk are various among mutations in different genes, and even the variants in the same gene have far different pathogenicity. For example, deficiencies of the natural anticoagulants were generally associated with tenfold or higher (especially AT deficiency) increased risk for VTE in heterozygous carriers, while factor V Leiden showed 5-fold increased risk in heterozygotes [7]. Prothrombin G20210A are correlated with ~ 2-4-fold risk for thrombosis in heterozygotes [8], while variants in prothrombin Arg596 residue (e.g. Arg596Leu, Arg596Gln) were correlated with high thrombotic risk and identified as significant pathogenic variants according to American College of Medical Genetics guidelines [9–11]. Mild or strong thrombogenic risks play different roles in VTE development and possibly influence the prevention and treatment of VTE.

Inherited PC deficiency, an important risk factor of hereditary thrombophilia in Asian population, was caused by numerous genetic alterations in *PROC* gene [12–14]. As reported, *PROC* (Arg189Trp (R189W) and Lys193del (K193del)) were considered to be hotspot mutants in Chinese population, with prevalent rates of 0.9% and 2.4% in general population and 5.21% and 6.52% in patients with VTE, respectively [15]. Several case-control studies revealed that R189W and K193del of PC had 5–8 folds and 2–3 folds higher predisposition to thrombosis, respectively [13, 16]. In 2013, we firstly revealed that R189W had normal amidolytic and proteolytic activities in the absence of cofactors but exhibited ~ 3 times lower affinity for binding to endothelial protein C receptor, while K193del had normal amidolytic and proteolytic activities in the absence of cofactors but exhibited ~ 2–3-fold impaired anticoagulant activity in the presence of PS *in vitro* [17]. Nevertheless, based on the long-term collection of clinical cases, we found that at least two-thirds of VTE patients with R189W or K193del mutations coexisting with other genetically or aquiredly prothrombotic risk factors. As VTE is a complex disease caused by the combination of environmental factors and genetic predisposition, a multifactorial analysis that includes the influence of genetic and environmental factors should be proposed for analyzing the independent contributions of R189W or K193del variants to VTE risk. Determination of the specific prothrombotic risk of *PROC* prevalent mutations (R189W or K193del) may influence future antithrombotic treatments, testing of other family members, and preventative measures to mitigate other risk factors for VTE.

In current study, we evaluated the independently prothrombotic risk of R189W and K193del mutations of PC in Chineses population after adjustment for age, gender and other inherited/acquried thrombotic risk factors.

## Methods

### Patients involvement

This study was approved by the ethics committee of Ruijin Hospital. A series of unrelated consecutive patients with a personal history of VTE who were registered at the Ruijin Hospital for thrombophilia screening from 2015 to 2022, were investigated. The inclusion criteria for patients with VTE were symptomatic deep vein thrombosis, pulmonary embolism, or thrombosis at other locations, which was confirmed objectively, regardless of age and period after the onset. Acquired high-risk situations, such as surgery, pregnancy, hormonal replacement therapy, oral contraceptives, or immobilization, were not exclusionary criterions. Healthy blood donors from Ruijin Hospital and Changhai Hospital who were selected and matched for age, sex and geographic region, without an individual or family history of VTE and arterial thrombosis, were enrolled in this study as well. Informed consent was obtained from all above participants. Data of their clinical performance, acquired risk factors of VTE, family history of VTE and laboratory examinations of probands as well as correlative family members were collected.

### Coagulation assays

Venous blood samples were collected in 0.109 mmol/L sodium citrate from all subjects. The whole blood sample was immediately centrifuged at 3000×g for 15 min to obtain the platelet-poor plasma. For the patients who were out of acute phase of VTE and had ceased anticoagulant therapy for at least two weeks, phenotypic analysis of thrombophilia were performed as previously described [18, 19], including chromogenic PC activity, AT activity, PS activity, plasminogen, lupus anticoagulant, anticardiolipin antibody, anti-β2 glycoprotein I, activity of coagulation factors and total homocysteine, etc. Above hemostatic tests for thrombophilia were performed in plasma-available family members if their corresponding probands had mutations with uncertain significance.

### The gene analysis in VTE patients and healthy controls

Genomic DNA was extracted from white blood cells using the QIAamp DNA blood purification kit (Qiagen, Hilden, Germany) according to manufacturer’s instructions and stored at -20℃ until use. The gene analysis using two high throughput technologies, which were massively parallel sequencing based on next generation sequencing for point variations and CNVplex® technique for copy number variations, was carried out in VTE patients and healthy controls. A total of 22 genes, including genes encoding natural anticoagulant proteins (*SERPINC1*, *PROS1* and *PROC*), coagulation factors (*F2, F5, F7, F8, F9, F10, F11, F12, F13A1*, *F13B, FGA, FGB, FGG* and *VWF*), fibrinolysis related proteins (*PLG, SERPINE1* and *SERPINF2*) and myeloproliferative disease related proteins (*JAK2* and *MPL*), were involved in our study. Variants were filtered to remove polymorphisms and benign variations as previous report [19]. Variants which had been reported with thrombotic risk were designated as prothrombotic variants. Novel variants including nonsense variants, frameshift variants and deletions in genes of the three natural anticoagulants that were consistent with identified impaired phenotype, were classified as prothrombotic variants. The pathogenicity of novel missense variants was determined through phenotypic results and bioinformatic analysis. Variants identified in genes without definite correlation to VTE were deemed to be uncertain significance. Only the mutation-corresponding sequence was detected in related family members.

### Statistical analysis

Statistical power was estimated by a priori power analysis using G*Power version 3.1.9.2 [20]. For continuous demographic, differences between case and control groups were assessed by a Student’s *t*-test or Mann-Whitney U-test, depending on the normality of the data. Categorical variables between the case and control groups were reported and their balance was assessed by chi-squared test. Unconditional logistic regressions was used to estimate crude odds ratios (ORs) of VTE with different demographic, PC R189W, PC K193del, other genetic and acquired factors. A multivariate logistic regression analysis was used to estimate the adjusted ORs of PC R189W and PC K193del after adjustment with age, gender, other inherited thrombotic risk factors (PC deficiency, PS deficiency, AT deficiency and other genetic risk factors) and acquried thrombotic risk factors (surgery, trauma, pregnancy/puerperium, sedentariness/immobilization and oral contraceptives et al.). Subgroup analysis was performed for the first-onset age and recurrence of VTE as well. The first-onset age subgroup was divided by the median of first-onset age. A two-tailed P < 0.05 was considered statistically significant. Analyses were performed using SPSS version 25.0 software (SPSS Inc., Chicago, IL).

## Results

### Baseline characteristics

A total of 490 patients and 410 controls, with mean (standard deviation, SD) ages of 39.8 (14.3 years; range 4–86.0) and 36.9 (13.5 years; range 4.0–81.0), respectively, were enrolled in the study. The number of participants according to gender (female: male) were 313:177 and 257:153 in patients and controls, respectively. In the group of VTE patients, the first-onset ages of VTE were 33.6 (13.8 years; range 3–82) and 36.0 (13.7 years; range 1–79) in males and females, respectively. 41% (201/490) patients suffered recurrent VTE.

**Table 1:** Frequencies of PC R189W and K193del in patients with history of VTE and in healthy controls in the Chinese cohort

Mutation	Genotype	Control group n (%)	VTE group n (%)	
PC R189W	Wide-type (R/R) Heterozygote (R/W)	407 (99.3) 3 (0.7)	463 (94.5) 27 (5.5)	
	Homozygote (W/W)	0 (0)	0 (0)	
PC K193del	Wide-type (K/K) Heterozygote (K/del)	403 (98.3) 7 (1.7)	447 (91.2) 41 (8.4)	
	Homozygote (del/del)	0 (0)	2 (0.4)	

PC, protein C; VTE, venous thromboembolism

### Clinical manifestations of patients with two hotspot ***PROC*** mutations

In current study, heterozygous p.R189W of PC was present in 27 VTE patients (5.5%, 27/410) and in 3 healthy controls (0.7%, 3/410), while no homozygote for this mutation was identified (Table 1). As shown in Suppl. Tables 1, 44.4% (12/27) patients with p.R189W suffered recurrent VTE and 25.9% (7/27) patients expierenced unusual sites of VTE. Among them, 25.9% (7/27) patients (patients 1–7) carried single PC R189W mutation without other inherited or acquired thrombotic risk factors but two of them suffered recurrent VTE. Besides, one patient (patient 8) with single PC R189W mutation suffered pulmonary embolism during her pregnancy. In addition, 55.6% (15/27) patients (patients 9–23) had another rare mutation of *PROC* aside from R189W, including 12 causative *PROC* mutations which had been reported before, and the other three novel mutations of *PROC* were associated with reduced PC activity that were confirmed through family segregation analysis. Among these patients, ten of them experienced multi-site and/or recurrent thrombotic episodes. Furthermore, 7.4% (2/27) patients (patients 24–25) had PC R189W mutation combined with causative *PROS1* heterozygotes. One patient (patient 26) had *PLG* defect resulted from large deletion of *PLG* gene. One patient (patient 27) had Klinefelter syndrome, which was a risk factor of VTE [21].

The K193del mutation of PC was identified in 8.8% (43/490) VTE patients and in 1.7% (7/410) controls. Two K193del homozygote was identified in the VTE group (0.4%) while the homozygous variant was absent in controls (Table 1). Their clinical manifestations, thrombophilic factors and family histories are shown in Suppl. Table 2. 46.5% (20/43) patients suffered recurrent VTE and 25.6% (11/43) patients expierenced unusual sites of VTE. There were 30.2% (13/43) patients (patients 28–40) had single PC K193del mutation without other inherited or acquired thrombotic risk factors. Four of them suffered recurrent VTE. We also identified 14.0% (6/43) patients (patients 41–46) had PC K193del mutation but developed VTE when they exposed to the acquired risk factors. Furthermore, 55.8% (24/43) patients (patients 47–70) with K193del combined with another mutation in gene encoding three natural anticoagulant proteins. 12 of them (patients 47–58) had another *PROC* mutation in which seven patients suffered recurrent VTE. Three patients (patients 48, 49 and 57) carried novel *PROC* mutations characterized by reduced PC chromogenic activity in corresponding affected patients. Besides, four different *SERPINC1* mutations were detected in four patients (patients 59–62) separately, in which two novel *SERPINC1* mutations had unclear pathogenicity while the other two mutations have been reported to be correlated with inherited AT deficiency. Moreover, *PROS1* mutations which had been stated with PS deficiency in previous studies were detected in other eight patients (patients 63–70) and five of them experienced recurrent VTE.

Therefore, in current study, 74% VTE patients carrying R189W mutation were combined with other risk factors of VTE, including 40.7% (11/27) patients with additional inherited risk factors of VTE, 3.7% (1/27) patients with acquired thrombotic risk factors and 29.6% (8/27) patients with both of them. Similar to R189W, 69.8% patients carrying PC K193del mutation were combined with other risk factors of VTE, including 46.5% (20/43) patients with other inherited risk factors of VTE, 14.0% (6/43) patients with acquired thrombotic risk factors and 9.3% (4/43) patients with both of them.

### Two hotspot ***PROC*** mutations and VTE risk

The crude odds ratio of developing VTE as a mutation carrier for PC R189W is 7.911 (95% confidence interval (CI), 2.382–26.272; *P* = 7.31** × **10^− 4^), and for PC K193del is 5.538 (95% CI, 2.382–12.450; *P* = 3.50** × **10^− 5^), as compared with individuals without above variants (Suppl. Table 3). Multivariate logistical regression analysis revealed that the association between these mutations and VTE were slightly decreased but still significant (the adjusted OR of R189W = 5.781; 95% CI 1.501–22.262; *P* = 0.011, and the adjusted OR of K193del = 4.365; 95% CI 1.743–10.931; *P* = 0.002) after adjustment for age, gender, inherited thrombotic risk factors (PC defect, PS defect, AT defect and other genetic risk factors) and acquired thrombotic risk factors (surgery, trauma, pregnancy/puerperium and sedentariness/immobilization, et al.) (Table 2). However, when compared with the odds ratios of rare mutation-induced PC defect (the adjusted OR = 84.479; 95% CI 11.499–620.654; *P* = 1.30** × **10^− 5^), PS defect (the adjusted OR = 79.009; 95% CI 19.111–326.631; *P* = 1.60** × **10^− 9^), AT defect (the adjusted OR = 61.318; 95% CI 8.258–455.326; *P* = 5.70** × **10^− 5^) and acquired thrombotic risk factors (the adjusted OR = 18.273; 95% CI 8.516–39.210; *P* = 8.77** × **10^− 14^), the contribution of PC R189W and K193del mutations to thrombosis were much less significant. Besides, advanced age was correlated with 1.021-fold (95% CI 1.009–1.033; *P* = 7.71** × **10^− 4^) increased risk of VTE, while gender was not.

In summary, the independent contribution of PC R189W and K193del mutations to VTE risk were much less significant compared with other rare mutations. However, due to the fact that most patients combined with other risk factors of thrombosis, the presence of R189W or K193del could amplify the thrombotic risk factor for 5.781-fold and 4.365-fold, respectively, resulting into remarkable increased thrombotic risk.

**Table 2:** Multivariate analysis of thrombotic risk in current cohort

Variants	VTE			First-onset of VTE			Recurrent VTE	
	Adjusted OR(95%CI)	*P* value		Adjusted OR(95%CI)	*P* value		Adjusted OR(95%CI)	*P* value
Gender	0.987 (0.699–1.396)	0.943		0.812 (0.536–1.229)	0.325		2.491 (1.586–3.913)	**7.40 × 10** ^**− 5**^
Age	1.021 (1.009–1.033)	**7.71 × 10** ^**− 4**^		/	/		1.038 (1.023–1.054)	**4.56 × 10** ^**− 7**^
PC R189W	5.781 (1.501–22.262)	**0.011**		0.986 (0.425–2.288)	0.974		0.971 (0.405–2.330)	0.947
PC K193del	4.365 (1.743–10.931)	**0.002**		0.867 (0.451–1.670)	0.670		1.804 (0.923–3.526)	0.085
Rare PC mutations	84.479 (11.499–620.654)	**1.30 × 10** ^**− 5**^		0.623 (0.374–1.038)	0.069		1.892 (1.103–3.246)	**0.020**
PS mutations	79.009 (19.111–326.631)	**1.60 × 10** ^**− 9**^		0.463 (0.289–0.743)	**0.001**		2.112 (1.256–3.553)	**0.005**
AT mutations	61.318 (8.258–455.326)	**5.70 × 10** ^**− 5**^		0.445 (0.232–0.851)	**0.014**		3.415 (1.723–6.769)	**4.33 × 10** ^**− 4**^
Other genetic mutations	2.35** × **10^9^	0.998		0.454 (0.171–1.205)	0.113		4.556 (1.656–12.533)	**0.003**
Acquired risk factors	18.273 (8.516–39.210)	**8.77 × 10** ^**− 14**^		0.375 (0.241–0.584)	**1.40 × 10** ^**− 5**^		2.107 (1.300–3.414)	**0.002**
Positive family history	/	**/**		1.365 (0.752–2.477)	0.307		1.731 (0.939–3.193)	0.079

AT, antithrombin; CI, confidence interval; OR, odds ratio; PC, protein C; PS, protein S; VTE, venous thromboembolism; *P* value < 0.05, significant difference

### Two hotspot ***PROC*** mutations and the first-onset age of VTE

**Fig. 1:**
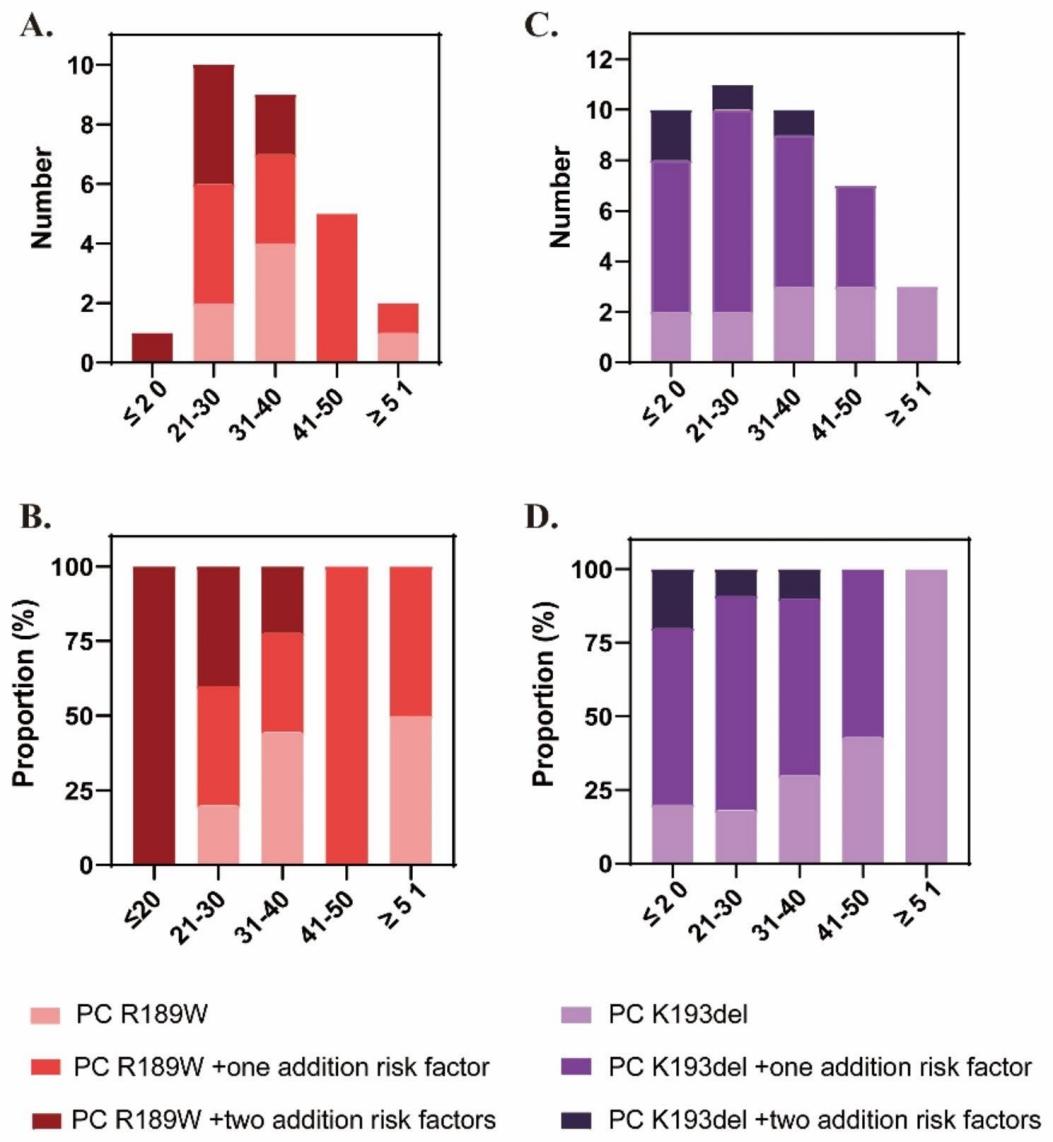
The first-onset age of VTE in patients with hotspot *PROC* mutations (R189W or K193del) independently or combined with additional risk factors. (**A**) number and (**B**) proportion of patients with PC R189W mutation in different age subsets. (**C**) number and (**D**) proportion of patients with PC K193del mutation in different age subsets

Multivariate analysis showed that rare mutations in *PROS1* and *SERPINC1* as well as environmental risk factors contributed to significant earlier onset age of VTE, whereas the R189W and K193del of PC were definitely not (adjusted ORs of R189W = 0.986; 95% CI 0.425–2.288; *P* = 0.974; adjusted ORs of K193del = 0.867; 95% CI 0.451–1.670; *P* = 0.670) (Table 2). As shown in Fig. 1, most patients with PC R189W or K193del suffered first episodes of VTE before 50 years old. Among the R189W carriers, seven out of them were free of other risk factors, 13 of them had one additional thrombotic risk factors and other seven patients had two additional risk factors. Their first-onset ages of VTE were 34.1 ± 12.7 years, 38.3 ± 10.0 years and 26.4 ± 6.6 years, respectively. Similar results were also found in patients with heterozygous K193del. 13 patients had single K193del mutation, 24 patients combined with one additional thrombotic risk factors and four patients combined with two additional risk factors. Their first-onset ages of VTE were 36.9 ± 14.5 years, 28.5 ± 11.2 years and 23.5 ± 7.72 years, respectively. Obviously younger first-onset ages of VTE were observed in patients with PC R189W or K193del mutation who combined with at least two additional risk factors of VTE than in those not.

Therefore, independent R189W and K193del of PC were not associated with early first-onset age of VTE. Combination of other genetic or acquired thrombophilic factors had supra-additive effects on the earlier onset age of VTE. The more additional risk factors the patients had, the younger first-onset ages of VTE might be.

### Two hotspot ***PROC*** mutations and the risk of recurrent VTE

**Fig. 2:**
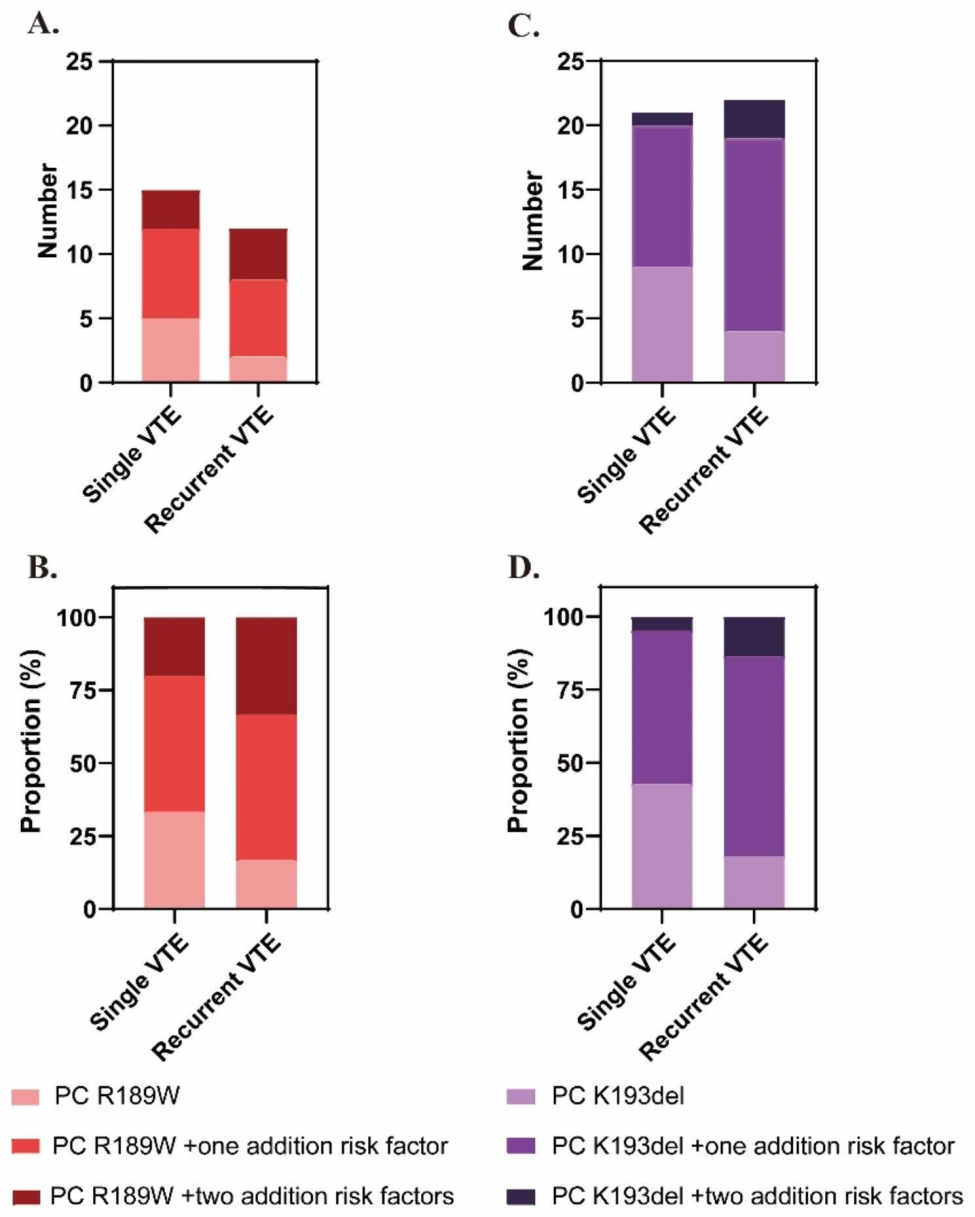
The recurrence of VTE in patients with hotspot *PROC* mutations (R189W or K193del) independently or combined with additional risk factors. (**A**) number and (**B**) proportion of patients with PC R189W mutation suffering from single or recurrent VTE episodes. (**C**) number and (**D**) proportion of patients with PC K193del mutation suffering from single or recurrent VTE episodes

As stated earlier, nearly half of patients with PC R189W or K193del mutation experienced recurrent VTE. Multivariate analysis demonstrated that advanced age, male, acquired risk factors, rare mutations in three natural anticoagulant, or rare mutations in other VTE-related genes were correlated with significant higher risk of VTE recurrence, whereas the R189W and K193del of PC were not (adjusted ORs of R189W = 0.971; 95% CI 0.405–2.330; *P* = 0.947; adjusted ORs of K193del = 1.804; 95% CI 0.923–3.526; *P* = 0.085) (Table 2). As shown in Fig. 2, R189W or K193del carriers without other thrombotic risk factors had few correlations with the recurrence of VTE. However, the proportions of R189W carriers with one additional risk factors (50.0% vs. 46.7%), and with two additional risk factors (33.3% vs. 20.0%) in recurrent-VTE group were higher than those in single-VTE group. The same tendencies were observed in patients with K193del mutation. The proportions of K193del carriers with one additional risk factors (66.7% vs. 50.0%), and with two additional risk factors (14.3% vs. 5.0%) in recurrent-VTE group were higher than those in single-VTE group. Taken together, coexisting of genetic or acquired thrombophilic factors had supra-additive effects on recurrence rate of VTE. The more additional risk factors the patients had, the higher risk of recurrence would be.

## Discussion

PC deficiency is an important risk factor of hereditary thrombophilia and two natural variants of *PROC* (R189W and K193del) have been identified as hotspot mutants in Chinese population [18]. Both R189 and K193 are located on a 21-residue peptide (residues 179–199) in the C terminus of the PC light chain, which connects the EGF2 domain (residues 135–178) to the activation peptide (residues 200–211) of the zymogen and is expected to contact with other macromolecules, such as activated factor V, thrombin-thrombomodulin complex, substrate, or phospholipid [16, 17, 22–24]. Our previous study showed that R189W of PC had normal amidolytic and proteolytic activities in the absence of cofactors but exhibited ~ 3 times lower affinity for binding to endothelial protein C receptor, while K193del of PC had normal amidolytic and proteolytic activities in the absence of cofactors but exhibited ~ 2–3-fold impaired anticoagulant activity in the presence of PS *in vitro* [17]. In 2020, Yamashita et al. stressed the functional importance of six residues within G184-L197 of PC (R185, K188, R189, K192, K193 and R194) on constituting activated protein C (APC)’s anticoagulant and cytoprotective activities [25]. According to their study, mutations at residue K192 or K193 exhibited 50% reduced APC’s anticoagulant activity with a decreased sensitivity for PS and reduced cytoprotective activities with an impaired ability to cleave PAR cleavage, while other residues including R189 presented similar APC’s anticoagulant activity and cytoprotective activities to wide-type APC [25].

In daily practice, diagnosis of PC deficiency is based on a determination of PC activity (PC:A), by either clot–based or mostly chromogenic assay. The current guidelines recommend the use of a chromogenic PC:A assay, as its less interferences, inter- and intralaboratory variations are expected, and it seems to be more cost-effective [26–28]. Based on the functional and immunological PC assays, PC deficiency can be divided into two types. Type I deficiency is featured by a parallel reduction in PC activity and in immunologically measured PC concentration, while type II deficiency is characterized by normal PC concentration with decreased PC activity. The latter can be subdivided further in type IIa deficiency with both reduced anticoagulant activity and amidolytic activity and, in type IIb deficiency, with reduced anticoagulant activity only [29, 30]. According to previous reports, both antigen and activity levels of PC in cases with heterozygous R189W were lower than those of healthy controls [16, 22, 31]. However, the PC:A level was significantly lower than the PC:Ag level (clot-based PC:A vs. PC:Ag: 59.8 ± 9.2% vs.71.7 ± 12.4% [22]; chromogenic PC:A vs. PC:Ag: 40.4–62.3 U/dl vs. 68.7–83.0 U/dl [16]) in the group of R189W heterozygotes themselves, indicating it’s a type II PC deficiency. In addition, it was reported that heterozygous state of K193del mutation showed normal PC:Ag and chromogenic PC:A, while the level of clot-based PC:A was controversial [13, 32]. The great majority of studies stated that heterozygous K193del was associated with lower anticoagulant activity of PC, suggesting it’s a type IIb PC deficiency, whereas some invesitigations demonstrated that the clot-based PC:A of heterozygotes K193del were within the reference interval [22, 32, 33]. The mutations related to inherited type IIb deficiency were possibly missed if DNA mutation analysis were only considered for those patients with decreased PC amidolytic activity, thereby stressing the importance of additional tests. Genetic analysis should be carried out directly even if patients had normal anticoagulant activities.

R189W of PC was firstly described in an American patient with symptomatic PC deficiency in 1995 [30] and subsequenctly identified as a common inherited thrombophilia in Asian population. In recent years, two independent case-control studies demonstrated that the prevalence rate of R189W in the healthy Chinese population and patients with VTE were 0.85–0.87% and 4.31–5.88%, respectively, indicating that the heterozygous R189W was a significant risk factor for VTE in Chinese population with 5.10–7.34 folds increased risk of VTE [16, 22, 32]. K193del of PC was first described in three Japanese patients who suffered from PC deficiency in 1998 [34], however, few large case-control study supported that this variant made a contribution to the higher risk for VTE in Japanese population. In 2012, Tang et al. firstly evaluated its prevalence in Chinese population (2.42% of healthy controls and 6.78% of patients with history of VTE) and determined that this variant was a risk factor for VTE in Chinese population with an odds ratio of 2.71 after adjustment for age, gender and some aquried thrombotic risk factors [32], but no inherited risk factors were considered in their dominant model. In 2020, Tsuda et al. assessed the distribution of PC R189W and K193del among East Asian subpopulations through *PROC* analysis in 2850 unrelated individuals from five countries (Japan, South Korea, Singapore, Hungary, and Brazil) [35]. However, due to the limited sample-size and complexity of ethnic composition of some subpopulations, data on the prevalence of R189W and K193del and their prothrombotic risk was limited and should be further evaluated [35].

In Table 3, we summarized the influence of two prevalent PC mutations on VTE in Asians population in previous reports. In our study, heterozygous R189W of PC was identified in 0.7% healthy controls and 5.5% patients with history of VTE, while K193del of PC was identified in 1.7% healthy controls and 8.8% patients with VTE episodes. However, due to the fact that most patients had R189W and K193del of PC coexisted with other prothrombotic risk factors, we decided to evaluate their ORs after adjustment for age, gender, inherited thrombotic risk factors (PC defect, PS defect, AT defect and other genetic risk factors) and acquired thrombotic risk factors. Multivariate logistical regression analysis revealed that R189W and K193del were associated with 5.781-fold (95% CI 1.501–22.262) and 4.365-fold (95% CI 1.743–10.931) increased risk of VTE, respectively, which were higher than heterozygous prothrombin G20210A (two to four-fold increased risk) but lower than heterozygous FV Leiden (10-fold increased risk) [36]. Meanwhile, we assessed the adjusted ORs of PC deficiency that resulted from other rare *PROC* mutations, PS deficiency, AT deficiency and acquired thrombotic risk factors in the same cohort. It can be seen, R189W or K193del mutation made not that significant contribution to the VTE development but may act in concert with other genetic defects or acquired thrombotic risk factors to produce the final severe phenotype.

Determination of the risk of recurrent VTE in heterozygous carriers of R189W or K193del is important for formulating optimal duration of anticoagulation therapy because that anticoagulant therapy varied with the phase of disease process, the number of thrombotic events (first versus recurrent) as well as the risk of VTE-onset or recurrence. However, few studies explicitly addressed this question. In current study, multivariate analysis revealed that the single R189W and K193del of PC were unassociated with earlier onset of first VTE episodes and higher risk of VTE recurrence. Combination of other genetic or acquired thrombophilic factors had supra-additive effects on the on overall thrombotic risk, characterized by earlier first-onset age of VTE and higher recurrence rate of VTE. Referring to the current guidelines of VTE management, we raise the following suggestions: first, the management of acute VTE episode, regardless of whether patients have inherited PC deficiency or not, requires sufficient anticoagulation [37]. The updated recommendations support the direct oral anticoagulants (i.e., rivaroxaban and apixaban) as the optimal treatment for most VTE patients [38]. Second, the duration of anticoagulation therapy must be personalized based on the risk of VTE recurrence and of bleeding complications. In general, 3–6 months of oral anticoagulation is recommended after a first VTE in mutation carriers, especially for those with transient risk factor (i.e. surgery, pregnancy/puerperium, hormonal therapy, immobilization, trauma), but a long-term prophylactic anticoagulation may be considered for those patients with a higher risk of VTE recurrence (i.e. patients with heterozygous R189W or K193del suffered unprovoked VTE, homozygous R189W or K193del carriers or the presence of other perpetually prothrombotic risk factors) [1]. Third, in view of the weak prothrombotic risk observed in our study, long-term anticoagulation is not routinely recommended for asymptomatic PC R189W or K193del heterozygotes, but a short course of prophylactic anticoagulation when acquired risk factors are present may prevent the patients from suffering initial thrombosis [39].

In conclusion, as the most frequent mutations for PC deficiency in Chinese population, both R189W and K193del mutations made not that significant contribution to VTE development but might essentially increase the thrombotic risk when combined with other prothrombotic mutations and/or under physiological conditions where additional underlying risk factors were presented.

**Table 3:** Influence of two prevalent PC mutations (R189W and K193del) on VTE in Asians population

	No.of deficiency		Odds ratio (95% confidence interval )	*P* value	Poplation	Reference (year)
	Cases, N (%)	Controls, N (%)				
PC R189W	^Het^ 5/116 (4.31)	^Het^ 11/1,292 (0.85)	^Het^ 5.10 (1.7–14.8)		Taiwanese Chinese	Tsay et al. (2004) [22]
	^Het^ 1/85 (1.2)	^Het^ 0/30 (0)			Japanese	Kinoshita et al. (2005) [40]
	^Het^ 59/1,003 (5.88)	^Het^ 9/1,031 (0.87)	^Het^ 7.10 (3.50–14.39), adjusted: 7.34 (3.61–14.94)	3.31*10^− 10^, adjusted:3.88*10^− 8^	Chinese	Tang et al. (2012) [16, 32]^a^
	^Het^ 68/1,304 (5.21)	^Het^ 12/1,334 (0.90)	^Het^ 6.06 (3.26–11.25)	1.03*10^− 10^	Chinese	Tang et al. (2013) [15]^a^
	^Het^ 18/184 (9.8) ^Hom^ 5/184 (2.7)	^Het^ 40/690 (5.8) ^Hom^ 1/690 (0.1)	^Het^ 1.8 (1.0–3.2) ^Hom^ 20.3 (2.3–173.7)	^Het^ 0.042 ^Hom^ <0.001	Thai Children	Sirachainan et al. (2018) [31]
	^Het^ 1/283(0.4)	^Het^ 0/868 (0)			Japanese	Tsuda et al. (2020) [35]
	^Het^ 16/83(19.3) ^Hom^ 1/83 (1.2)	^Het^ 2/74(2.7) ^Hom^ 0/74 (0)			Singapore	Tsuda et al. (2020) [35]
	^All^ 0/85 (0)	^All^ 0/140 (0)			South Korea	Tsuda et al. (2020) [35]
PC K193del	^Het^ 2/85 (2.4)	^Het^ 1/30 (3.3)			Japanese	Kinoshita et al. (2005) [40]
	^Het^ 4/173 (2.3)				Japanese	Miyata et al. (2009) [13]
	^Het^ 68/1,003 (6.78) ^Hom^ 1/1,003 (0.09)	^Het^ 25/1,031 (2.42) ^Hom^ 0/1,031 (0)	^Het^ 2.88 (1.81–4.60) adjusted: 2.71(1.68–4.36) ^All^ 2.93 (1.84–4.67) adjusted: 2.71 (1.68–4.38)	^Het^ 3.80*10^− 6^ adjusted: 4.04*10^− 5^ ^All^ 2.59*10^− 6^ adjusted: 4.59*10^− 5^	Chinese	Tang et al. (2012) [32]^a^
	^All^ 85/1,304 (6.52)	^All^ 32/1,334 (2.40)	^All^ 2.84 (1.88–4. 29)	^All^ 2.77*10^− 7^	Chinese	Tang et al. (2013) [15]^a^
	^All^ 9/100 (4.5)	^All^ 5/268 (0.9)	^All^ 5.00 (1.66–15.12) adjusted:5.34, (1.47–19.39)	^All^ 0.004 adjusted: 0.011	Chinese	Wang et al. (2016) [41]
	^Het^ 4/446 (0.9) ^Hom^ 2/446 (0.4)	^Het^ 7/968 (0.7) ^Hom^ 0/968 (0)			Japanese	Tsuda et al. (2020) [35]
	^Het^ 6/83 (7.2) ^Hom^ 0/83 (0)	^Het^ 0/74 (0) ^Hom^ 0/74 (0)			Singapore	Tsuda et al. (2020) [35]
	^Het^ 2/85 (2.5) ^Hom^ 1/85 (1.2)	^Het^ 1/140 (0.7) ^Hom^ 1/140 (0.7)			South Korea	Tsuda et al. (2020) [35]

PC, protein C. ^a^ The recruiment of subjects in these two case-control studies largely overlapped. ^All^, heterozygous and homozygous; ^Het^, heterozygous; ^Hom,^ homozygous

### Electronic supplementary material

Below is the link to the electronic supplementary material.

Supplementary Material 1

## Data Availability

The datasets used and/or analysed during the current study are available from the corresponding author on reasonable request.

## List of abbreviations

APC: Activated protein C
APS: Antiphospholipid antibody syndrome
AT: Antithrombin
CI: Confidence interval
OR: Odds ratio
PC: Protein C
PC:A: PC activity
PS: Protein S
SD: Standard deviation
VTE: Venous thromboembolism
