# Supplementary material for: Evaluation of prothrombotic risk of two PROC hotspot mutations (Arg189Trp and Lys193del) in Chinese population: a retrospective study
Source: Thromb J. 2023 Oct 3;21:103. doi: 10.1186/s12959-023-00548-6 (PMC10546776; doi:10.1186/s12959-023-00548-6)
Supplement: Supplementary file 1 — Supplementary Material 1 [file 12959_2023_548_MOESM1_ESM.docx]

**Suppl. Table 1. Clinical manifestations, phenotype and genotype results of 27 unrelated pedigrees with PC R189W variant.**

| **No.** | **Age/ sex** | **Thrombosis episode**  **(onset age)** | **Acquired VTE factors** | **Combined other genetic defects** | **Genetic variants** |
| --- | --- | --- | --- | --- | --- |
| 1 | 29/M | PE (27)(29) |  |  | **PC: R189W** ^MI^ |
| 2 | 34/M | BL-DVT and PE (32) |  |  | **PC: R189W** ^FI^ |
| 3 | 38/M | PVT (31)(35) |  |  | **PC: R189W** ^FI^ |
| 4 | 26/F | RL-DVT and PE (21) |  |  | **PC: R189W** ^FI^ |
| 5 | 65/F | RL-DVT and PE (61) |  |  | **PC: R189W** |
| 6 | 32/M | CVT (31) |  |  | **PC: R189W** ^MI^ |
| 7 | 37/F | LL-DVT (36) |  |  | **PC: R189W** |
| 8 | 27/F | PE (26) | Pregnant |  | **PC: R189W** |
| 9 | 48/M | LL-DVT, PE (48) |  | PC defect | **PC: R189W** ^FI^; V26M ^MI^ |
| 10 | 54/M | LL-SUVT (34); RL-DVT (50) |  | PC defect | **PC: R189W**; W83R |
| 11 | 35/M | BL-DVT (29) |  | PC defect (PC: A 53%) | **PC: R189W** ^MI^; c.262+2T>C ^FI^ † |
| 12 | 49/M | PE (47) |  | PC defect | **PC: R189W**; G109R |
| 13 | 24/M | LL-DVT (14)(22)  RL-DVT (23) | APS | PC defect | **PC: R189W**; F181V ^MI^ |
| 14 | 38/M | BL-DVT (34) | Surgery | PC defect | **PC: R189W** ^FI^; **K193del** ^MI^ |
| 15 | 54/M | LL-DVT (48)  RL-DVT, PE (53) |  | PC defect | **PC: R189W**; **K193del** |
| 16 | 33/M | LL-DVT (23)  BL-DVT (24)(31) | Trauma 1^st^ | PC defect | **PC: R189W** ^MI^; R211Q ^FI^ |
| 17 | 56/M | BL-DVT (49)(54) |  | PC defect | **PC: R189W**; R220W |
| 18 | 26/M | CVT (21) |  | PC defect | **PC: R189W**; R220W |
| 19 | 34/F | PVT (31) | OC | PC defect (PC: A 58%) | **PC: R189W**; N361D † |
| 20 | 31/M | RL-DVT (27)(30) | Sedentariness 1^st^ | PC defect (PC: A 49%) | **PC: R189W** ^MI^; E372G ^FI^ † |
| 21 | 33/M | LL-DVT and PE (29) |  | PC defect | **PC: R189W** ^FI^; M377T ^MI^ |
| 22 | 69/M | RL-DVT (51); LL-DVT (60)  UE-DVT (66) | Surgery 2^nd^ | PC defect (PC: A 45%) | **PC: R189W**; M380MfsX41† |
| 23 | 58/M | LL-DVT and PE (42)  RL-DVT (56) |  | PC defect | **PC: R189W**; L386P ^MI^ |
| 24 | 35/M | MI (26)  PE, RL-DVT (44) | Sedentariness 1^st^ | PS defect | **PC: R189W**; PS: E67A |
| 25 | 38/M | PVT (36） |  | PS defect | **PC: R189W** ^FI^; PS: R561W ^MI^ |
| 26 | 46/M | PE (38) CI (40) |  | PLG defect | **PC: R189W**; PLG: exon1-2 deletion |
| 27 | 32/M | LL-DVT (30) | Hyperlipidemia | Klinefelter syndrome | **PC: R189W** |

APS, anti-phospholipid syndrome; BL-DVT, bilateral leg deep venous thrombosis; CI, cerebral infarction; CVT, cerebral venous thrombosis; F, female; LL-DVT, left leg deep venous thrombosis; LL-SUVT, left leg superficial vein thrombosis; M, male; MI, myocardial infarction; OC, oral contraceptives; PC, protein C; PC: A, protein C chromogenic activity; PE, pulmonary embolism; PLG, plasminogen; PS, protein S; PVT, portal venous thrombosis; RL-DVT, right leg deep venous thrombosis; UE-DVT, upper extremity deep venous thrombosis. †, Novel mutation; ^MI^, variant inherited from mother; ^FI^, variant inherited from father. Note: The numbering of changed amino acid residue was numbered by regarding the translation initiation site (start codon) as residue 1. The protein C activity listed within brackets were associated with corresponding novel *PROC* mutation. Reference range: PC: A, 60–130%.

**Suppl. Table 2. Clinical manifestations, phenotype and genotype results of 43 unrelated pedigrees with PC K193del variant.**

| **No.** | **Age/ sex** | **Thrombosis episode**  **(onset age)** | **Acquired VTE factors** | | **Combined other genetic defects** | | **Genetic variants** |
| --- | --- | --- | --- | --- | --- | --- | --- |
| 28 | 59/F | PE (53) |  | |  | | **PC: K193del** |
| 29 | 22/M | LL-DVT (16) |  | |  | | **PC: K193del** ^MI^ |
| 30 | 50/F | LL-DVT and PE (46) |  | |  | | **PC: K193del** |
| 31 | 48/M | CVT (43) |  | |  | | **PC: K193del** ^FI^ |
| 32 | 30/M | RL-DVT (25) |  | |  | | **PC: K193del** |
| 33 | 47/M | LL-DVT (38); PE (42) |  | |  | | **PC: K193del** |
| 34 | 52/F | PE (45)(52); LL-DVT (52) |  | |  | | **PC: K193del** |
| 35 | 26/F | CVT (23) |  | |  | | **PC: K193del** ^MI^ |
| 36 | 54/M | LL-DVT and PE (51) |  | |  | | **PC: K193del** |
| 37 | 19/M | LL-DVT (14) |  | |  | | **PC: K193del** |
| 38 | 64/M | RL-DVT and PE (60)(63) |  | |  | | **PC: K193del** |
| 39 | 41/M | CVT (31); CVT and PE (38) |  | |  | | **PC: K193del** ^FI^ |
| 40 | 35/M | RL-DVT and PE (35) |  | |  | | **PC: K193del** |
| 41 | 47/F | BL-DVT (43) (47) | Surgery ^1st^ | |  | | **PC: K193del** ^FI^ |
| 42 | 28/F | CVT (26) | Pregnant | |  | | **PC: K193del** |
| 43 | 58/F | LL-DVT (25)(56) | Puerperium 2^nd^ | |  | | **PC: K193del** |
| 44 | 36/F | SA (28, 30,31); PE (32) | IVF 4^th^ | |  | | **PC: K193del** ^MI^ |
| 45 | 59/M | RL-DVT (51)(57)  CVT (52) | Sedentariness 1^st^  Surgery 3^rd^ | |  | | **PC: K193del** ^Hom^ |
| 46 | 38/F | JVT (37) | Pregnancy 1^st^ | |  | | **PC: K193del** |
| 47 | 15/M | LL-DVT and PE (13) |  | | PC defect | | **PC: K193del**; c.-1535A>G ^MI^ |
| 48 | 17/M | RL-DVT (14) |  | PC defect (PC: A 52%) | | **PC: K193del** ^MI^; C101W ^FI^ † | |
| 49 | 33/M | LL-DVT (19); PVT (31)  CVT and UE-DVT (33) |  | PC defect (PC: A 42%) | | **PC: K193del** ^MI^: G165C ^FI^ † | |
| 50 | 34/M | PE (33) |  | PC defect | | **PC: K193del** ^MI^; F181V ^FI^ | |
| 51 | 38/M | BL-DVT (34) | Surgery 1^st^ | PC defect | | **PC: K193del** ^MI^; **R189W** ^FI^ | |
| 52 | 54/M | LL-DVT (48)  RL-DVT and PE (53) |  | PC defect | | **PC: K193del**; **R189W** | |
| 53 | 48/M | RL-DVT (38)(45); LL-AT (43) |  | | PC defect | | **PC: K193del**; c.678+9C>T |
| 54 | 32M | LL-DVT (21); RL-DVT (28) |  | | PC defect | | **PC: K193del**; D299AfsX16 |

**Suppl. Table 2: Continued**

| **No.** | **Age/ sex** | **Thrombosis episode**  **(onset age)** | **Acquired VTE factors** | **Combined other genetic defects** | **Genetic variants** |
| --- | --- | --- | --- | --- | --- |
| 55 | 27/M | PE (21); LL-DVT (23) |  | PC defect | **PC: K193del** ^MI^; V367M ^FI^ |
| 56 | 25/M | LL-DVT (20)(25) |  | PC defect | **PC: K193del** ^MI^; V339M ^FI^ |
| 57 | 36/M | LL-DVT (32) |  | PC defect (PC: A 40%) | **PC: K193del** ^MI^; D451EfsX54 ^FI^ † |
| 58 | 46/M | MI (43); PVT (45) |  | PC defect | **PC: K193del**; Q456RfsX49 ^MI^ |
| 59 | 30/M | CVT (23); RL-DVT (27) |  | AT defect (AT: A 97%) | **PC: K193del** ^FI^; AT: K289E ^FI^ † |
| 60 | 42/M | RL-DVT and PE (39); PE (40) |  | AT defect (AT: A 90%) | **PC: K193del** ^MI^; AT: M313T † |
| 61 | 22/M | CVT (11)(17);  RL-DVT and PE (14) |  | AT defect | **PC: K193del** ^MI^; AT: 297delE |
| 62 | 21/M | RL-DVT (21) |  | AT defect | **PC: K193del**; AT: A436D |
| 63 | 16/M | LL-DVT (14) |  | PS defect | **PC: K193del** ^Hom^; PS: c.76+1G>A |
| 64 | 35/M | BL-DVT, PE (30)(33)(34) |  | PS defect | **PC: K193del** ^FI^; PS: L45X ^MI^ |
| 65 | 23/M | RL-DVT (15) |  | PS defect | **PC: K193del**; PS: E67A |
| 66 | 28/M | RL-DVT (20); LL-DVT (27) | APS | PS defect | **PC: K193del** ^FI^; PS: E67A ^FI^ |
| 67 | 35/M | LL-DVT and PE (16)  CVT, LL-DVT and SUVT (32) | APS | PS defect | **PC: K193del**; PS: M251VfsX17 ^MI^ |
| 68 | 26/F | RL-DVT (24)(25); PE (26) | OC 1^st^; Pregnancy 2^nd^ ; Surgery 3^rd^ | PS defect | **PC: K193del** ^MI^; PS: Y560X ^MI^ |
| 69 | 39/M | RL-DVT (38) |  | PS defect | **PC: K193del**; PS: R561W |
| 70 | 52/F | BL-DVT (47); PVT (51) |  | PS defect | **PC: K193del**; PS: exon1-4 deletion |

APS, anti-phospholipid syndrome; AT, antithrombin; BL-DVT, bilateral leg deep venous thrombosis; CVT, cerebral venous thrombosis; F, female; IVF, in vitro fertilization; JVT, jugular vein thrombosis; LL-AT, left leg arterial thrombosis; LL-DVT, left leg deep venous thrombosis; M, male; MI, myocardial infarction; ND, no detection; OC, oral contraceptives; PC, protein C; PC: A, protein C chromogenic activity; PE, pulmonary embolism; PS, protein S; PVT, portal venous thrombosis; RL-DVT, right leg deep venous thrombosis; SA, spontaneous abortion; SUVT, superficial vein thrombosis; UE-DVT, upper extremity deep venous thrombosis. ^hom^, homozygous mutation; †, Novel mutation; ^MI^, variant inherited from mother; ^FI^, variant inherited from father. Note: The numbering of changed amino acid residue was numbered by regarding the translation initiation site (start codon) as residue 1. The protein C activity or antithrombin activity listed within brackets were associated with corresponding novel *PROC* or *SERPINC1* mutation. Reference range: PC: A, 60–130%; AT: A, 84.6–120.2%.

**Suppl. Table 3. Univariate analysis of thrombotic risk in current cohort**

| **Mutations** | **VTE** | |  | **First-onset of VTE** | |  | **Recurrent VTE** | |
| --- | --- | --- | --- | --- | --- | --- | --- | --- |
|  | **Crude OR（95%CI）** | ***P* value** |  | **Crude OR（95%CI）** | ***P* value** |  | **Crude OR（95%CI）** | ***P* value** |
| Gender | 1.053 (0.802-1.382 ) | 0.711 |  | 0.899 (0.621-1.300) | 0.571 |  | 2.269 (1.533-3.359) | **4.20*10^-5^** |
| Age | 1.009 (1.000-1.019) | 0.056 |  | / | **/** |  | 1.025 (1.012-1.038) | **2.02*10^-4^** |
| PC R189W | 7.911 (2.382-26.272) | **7.31*10^-4^** |  | 0.923 (0.423-2.015) | 0.840 |  | 1.129 (0.517-2.466) | 0.761 |
| PC K193del | 5.538 (2.463-12.450) | **3.50*10^-5^** |  | 0.910 (0.485-1.708) | 0.768 |  | 1.525 (0.815-2.856) | 0.187 |
| other *PROC* mutations | 78.616 (10.886-567.729) | **1.50*10^-5^** |  | 0.754 (0.462-1.230) | 0.258 |  | 1.545 (0.953-2.504) | 0.078 |
| *PROS1* mutations | 54.295 (13.306-221.542) | **2.58*10^-8^** |  | 0.551 (0.351-0.866) | **9.74*10^-3^** |  | 1.741 (1.124-2.697) | **0.013** |
| *SERPINC1* mutations | 44.426 (6.103-323.255) | **1.80*10^-4^** |  | 0.547 (0.292-1.026) | 0.06 |  | 2.567 (1.388-4.747) | **0.003** |
| Other genetic mutations | 1.41*10^9^ | 0.998 |  | 0.666 (0.258-1.720) | 0.401 |  | 2.491 (0.963-6.442) | 0.060 |
| Acquired risk factors | 17.209 (8.304-35.663) | **1.96*10^-14^** |  | 0.387 (0.251-0.599) | **2.00*10^-5^** |  | 1.190 (0.790-1.793) | 0.405 |
| Positive family history | / | **/** |  | 0.948 (0.555-1.618) | 0.844 |  | 2.499 (1.446-4.317) | **0.001** |

AT, antithrombin; CI, confidence interval; OR, odds ratio; PC, protein C; PS, protein S; VTE, venous thromboembolism; *P* value <0.05, significant difference.
